# Efficacy of Two *Chlamydia abortus* Subcellular Vaccines in a Pregnant Ewe Challenge Model for Ovine Enzootic Abortion

**DOI:** 10.3390/vaccines9080898

**Published:** 2021-08-13

**Authors:** Morag Livingstone, Sean Ranjan Wattegedera, Javier Palarea-Albaladejo, Kevin Aitchison, Cecilia Corbett, Michelle Sait, Kim Wilson, Francesca Chianini, Mara Silvia Rocchi, Nicholas Wheelhouse, Gary Entrican, David Longbottom

**Affiliations:** 1 Moredun Research Institute, Pentlands Science Park, Midlothian EH26 0PZ, UK; morag.livingstone@moredun.ac.uk (M.L.); sean.wattegedera@moredun.ac.uk (S.R.W.); kevin.aitchison@moredun.ac.uk (K.A.); Cecilia.Corbett@apha.gov.uk (C.C.); michelle.sait@unimelb.edu.au (M.S.); kim.wilson@ed.ac.uk (K.W.); francesca.chianini@moredun.ac.uk (F.C.); mara.rocchi@moredun.ac.uk (M.S.R.); n.wheelhouse@napier.ac.uk (N.W.); Gary.Entrican@roslin.ed.ac.uk (G.E.); 2 Biomathematics and Statistics Scotland, Edinburgh EH9 3FD, UK; javier.palarea@udg.edu

**Keywords:** *Chlamydia abortus*, enzootic abortion of ewes, vaccine development, vaccine efficacy, quantitative real-time polymerase chain reaction (PCR), serological analysis, cytokine analysis

## Abstract

*Chlamydia abortus*, the aetiological agent of enzootic abortion of ewes, is a major cause of reproductive loss in small ruminants worldwide, accounting for significant economic losses to the farming industry. Disease can be managed through the use of commercial inactivated or live whole organism-based vaccines, although both have limitations particularly in terms of efficacy, safety and disease-associated outbreaks. Here we report a comparison of two experimental vaccines (chlamydial outer membrane complex (COMC) and octyl glucoside (OG)-COMC) based on detergent extracted outer membrane preparations of *C. abortus* and delivered as prime-boost immunisations, with the commercial live vaccine Cevac^®^ Chlamydia in a pregnant sheep challenge model. No abortions occurred in either experimental vaccine group, while a single abortion occurred in the commercial vaccine group. Bacterial shedding, as a measure of potential risk of transmission of infection to naïve animals, was lowest in the COMC vaccinated group, with reductions of 87.5%, 86.4% and 74% observed for the COMC, OG-COMC and live commercial vaccine groups, respectively, compared to the unvaccinated challenge control group. The results show that the COMC vaccine performed the best and is a safer efficacious alternative to the commercial vaccines. However, to improve commercial viability, future studies should optimise the antigen dose and number of inoculations required.

## 1. Introduction

Enzootic abortion of ewes (EAE), also known as ovine enzootic abortion (OEA) or ovine chlamydial abortion, is caused by the obligate intracellular, Gram-negative bacterium *Chlamydia abortus*. Although the disease occurs mainly in sheep and goats, the pathogen can also cause abortion in a wide range of other animals, including cattle, pigs, deer and horses [1,2,3,4,5]. The pathogen has a global distribution, with the notable exceptions of New Zealand and Australia, and is one of the most common infectious causes of abortion worldwide [6]. In the UK, EAE is consistently the most commonly diagnosed infectious cause of ovine fetopathy, accounting for 43–45% of cases [7]. Importantly, *C. abortus* is a zoonotic pathogen and as well as leading to abortion or still-birth of the unborn child, it has the potential to cause serious pathology in pregnant women and is therefore a potential significant health risk for those in contact with sheep, especially at lambing [8,9,10,11,12].

Infected animals generally lack any clinical symptoms, with the first signs of a problem being the pre-term delivery of one or more dead lambs or the birth of weak offspring that do not survive [4,13]. Often, sheep deliver a combination of dead, weak and apparently normal healthy lambs. Animals can shed high loads of *C. abortus* in products of abortion, vaginal excretions and on the coats of live and dead lambs, all of which act as the main sources of transmission to naïve sheep [4,13].

Management of the disease in sheep in Europe has been achieved through the use of inactivated whole organism-based vaccines [14,15,16,17] or live attenuated vaccines based on the 1B strain of *C. abortus* (Cevac^®^ Chlamydia, Ceva Animal Health; Enzovax^®^, MSD Animal Health) [18,19]. However, both types of vaccines have limitations principally in terms of safety, stability, shedding of infectious organisms at parturition or in the duration of the protective immune response [1,20]. In addition, the live vaccines have also been implicated in causing infection or disease in some animals [21,22,23], while whole genome sequencing and comparative analysis have shown that there is no genetic basis for any attenuation in the vaccine strain [24]. Furthermore, the live vaccine has also been linked to the introduction of *C. abortus* infection into sheep flocks with no previous history of chlamydial abortion suggesting transmission from vaccinated to naïve animals [25].

Research into developing new EAE vaccines has focussed on evaluating alternate inactivated formulations [14,15,16,17,26], on virulence-associated recombinant antigens [27,28,29] and on the major outer membrane protein (MOMP) [30,31], which is also the major immunodominant antigen of *C. abortus*, with mixed results. In general, experimental chlamydial vaccines based on denatured or non-native recombinant MOMP preparations have yielded inconsistent results and only partial protection [20,32,33], suggesting that protein conformation may be important. Indeed, vaccine preparations based on chlamydial outer membrane complexes (COMCs), which are highly enriched for the MOMP in its native form, have been more promising and shown to be protective against chlamydial disease in sheep [34], guinea pigs [35], and mice [36,37], showing that conformation of MOMP, as well as other antigens on the chlamydial surface, may be important in eliciting the required protective immune response [20,38].

The aim of this study was to investigate the efficacy of two subcellular vaccines based on detergent-extracted whole organism *C. abortus* outer membrane preparations in pregnant ewes experimentally challenged with *C. abortus*, with a view to developing a safer and more stable alternative to the commercial live vaccines. Protective efficacy was assessed by reductions in adverse pregnancy outcomes, placental gross pathology, placental *C. abortus* load and shedding of *C. abortus* in vaginal excretions, following the delivery of dead and live lambs.

## 2. Materials and Methods

### 2.1. Ethics Statement

This study was carried out in strict accordance with the Animals (Scientific Procedures) Act 1986, in compliance with all UK Home Office Inspectorate regulations and ARRIVE guidelines. The experimental protocol was approved by the Moredun Animal Welfare Ethical Review Body (Permit number: E30/11; approved on 21 June 2011). All animals were monitored throughout the study for any clinical signs at least three times daily and all findings recorded. In the last four weeks of expected parturition monitoring increased to 24 h per day. Any animal found to be suffering or requiring treatment, for example from secondary bacterial infections, was given appropriate veterinary care (including use of antibiotics by a registered veterinary practitioner) in accordance with standard veterinary practice. All lambs born weak as a result of the challenge infection were independently assessed by a registered veterinary practitioner who took the decision to euthanise to end suffering based on the condition of the animal (criteria included not being able to stand or lift head but lying flat out on its side, not able or having no interest in suckling, not opening eyes, laboured respiration, general minimal signs of life) by administration of an overdose of sodium pentobarbital (Euthatal; Merial Animal Health Ltd., Harlow, Essex, UK). All ewes and lambs were continually monitored and given appropriate veterinary care, where required, following parturition, at least three times a day until the end of the experiment, which was 2 months post lambing.

### 2.2. Preparation of C. abortus Elementary Bodies (EBs)

*C. abortus* strain S26/3, which was isolated at the Moredun Research Institute in Scotland in 1979 from a vaccinated ewe that aborted, was propagated in McCoy cells in accordance with previously published protocols [39,40]. Infected cells were harvested using sterile glass beads at 72 h post infection, and EBs were purified by density gradient centrifugation through urografin, as described previously [39]. Purified EBs were resuspended in PBS and quantified using a BCA Protein Assay kit (Thermo Scientific™ Pierce™ BCA Protein Assay Kit, Fisher Scientific, Loughborough, UK) and stored at −20 °C until use.

### 2.3. Vaccine Antigen Preparation and Quantification

Two antigen preparations were produced from purified EBs for formulation into experimental Vaccines 2 and 3 for comparison with the live commercial vaccine (Vaccine 1: Cevac^®^ Chlamydia, Ceva Animal Health Ltd., Amersham, UK). For Vaccine 2, COMCs were prepared from EBs, as previously described [41], by incubating in phosphate buffered saline (PBS), pH 7.4, containing 10 mM EDTA and 2% sarcosyl (sodium N-lauroylsarcosine; Sigma-Aldrich Company Ltd., Gillingham, Dorset, UK) for 60 min at 37 °C with occasional mixing and sonication (5 s bursts) to prevent aggregation. Following centrifugation at 100,000× *g* for 60 min the pellet was resuspended in the same solution containing 10 mM dithiothreitol (DTT; Sigma-Aldrich Company Ltd., Gillingham, Dorset, UK) and incubated at 37 °C for a further 60 min. The mixture was centrifuged as before and washed in PBS before final sonication and resuspension of the insoluble COMC in PBS. For Vaccine 3, the insoluble COMC preparation (as produced for Vaccine 2) was extracted further by solubilising in 2% (*w*/*v*) n-Octyl Glucoside (OG)/10 mM DTT/PBS for 2 h at 37 °C. The mixture was centrifuged at 100,000× *g* for 60 min and the soluble fraction (OG-COMC) retained. Both antigen preparations were stored at −80 °C until formulated into the vaccines on the day of use.

Samples of the COMC and OG-COMC preparations were subjected to sodium dodecyl sulphate-polyacrylamide gel electrophoresis (SDS-PAGE) and the MOMP protein band quantified against a series of bovine serum albumin standards using ImageQuant TL 1D gel analysis software (GE Healthcare, Chicago, IL, USA) to give an estimate of MOMP concentration in each sample.

### 2.4. Formulation of Vaccine Preparations

Vaccines 2 and 3 were adjuvanted with Montanide^TM^ ISA 70 VG (Seppic SA, Paris, France) [42], following manufacturer’s instructions, using a ratio of adjuvant/antigen of 70/30 (weight/weight) containing a final concentration of 10 µg MOMP protein (prepared in PBS) per 1 mL. A stable emulsion was achieved using an Ultra Turrax homogeniser (IKA^®^-Werke GmbH and Co. K G, Staufen, Germany) at high shear rate (4000 rpm) for 3 min. Vaccine 1 was reconstituted in accordance with manufacturer’s instructions and used within 2 h. Vaccines 2 and 3 were stored at 4 °C for 1 month prior to use to ensure stability.

### 2.5. Preparation of C. abortus Challenge Inoculum

*C. abortus* strain S26/3 was grown in fertile hens’ eggs for preparing challenge inoculum, as previously described [43,44]. Briefly, infected yolk sacs were ground up using sterile sand in a mortar and pestle and suspended in PBS. Following centrifugation at 5000× *g* for 10 min to remove gross debris, the middle layer was carefully removed, aliquoted and stored in liquid nitrogen. The *C. abortus* EB titre in the yolk sac material was determined by inoculating 10-fold dilutions of the thawed stored material onto coverslips of McCoy cell monolayers, growing at 37 °C under CO_2_ for 72 h and counting the number of infected cells, as previously described [45]. Inoculum was diluted in PBS to 10^6^ inclusion-forming units (IFU) of *C. abortus* per mL.

### 2.6. Experimental Design

**Figure 1:**
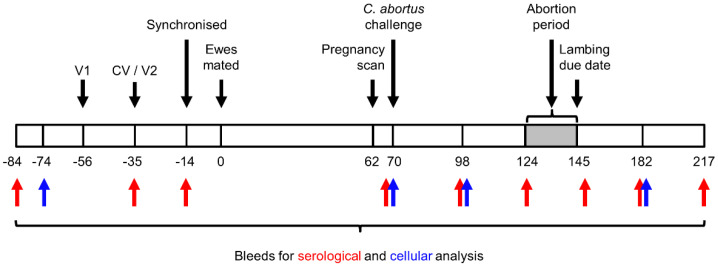
Experimental design. Numbers under bar indicate days prior to or post mating. V1 and V2 indicate timings for first and second vaccinations, respectively. CV indicates when the commercial vaccine was administered.

A flock of Scotch Mule sheep (crossbred sheep of Scottish Blackface ewes sired by Bluefaced Leicester rams; aged 3–5 years) were screened by rOMP90-3 Enzyme-linked immunosorbent assay (ELISA) [46] to ensure all animals were seronegative for *C. abortus*. Seronegative animals (*n* = 130) were randomly assigned to 5 groups, each containing 26 ewes. Groups 1–3 were vaccinated as follows: group 1 animals received a single 2 mL dose of the commercial Vaccine 1, in accordance with the manufacturer’s instructions, five weeks before mating; group 2 and 3 animals were vaccinated twice each with a 1 mL dose of the COMC Vaccine 2 and OG-COMC Vaccine 3 preparations (both containing 10 µg MOMP per 1 mL dose), respectively, at eight and five weeks prior to mating. All initial vaccines were administered intramuscularly (i.m.) on the left side of the neck, while second vaccine doses in group 2 and 3 animals were administered i.m. on the right side of the neck. Group 4 and 5 animals were not vaccinated and served as positive and negative controls, respectively. Three weeks after the second vaccinations, all ewes were synchronised using progesterone sponges (Veramix, Upjohn Ltd., Crawley, UK) over two weeks and then mated. At day 70 of gestation, all pregnant vaccinated ewes (groups 1–3) and group 4 positive control ewes were inoculated subcutaneously (s.c.) over the left prefemoral lymph node with 2 mL of challenge inoculum containing 2 × 10^6^ IFU of *C. abortus*. Group 5 animals served as unvaccinated and non-challenged negative controls and were housed separately from the other groups. All animals were fed on a normal maintenance diet with free access to hay and water. The clinical outcome of each ewe was recorded, along with the weight and sex of each lamb/foetus immediately after delivery. A ewe was considered to have aborted if they delivered at least one dead lamb, or a weak lamb that had to be euthanised on animal welfare grounds or died within 48 h of birth and when chlamydial EBs/DNA were demonstrable in the foetus, placenta or uterine discharges by stained smears, real-time PCR or through pathological investigation. A summary of the experimental design is depicted in Figure 1.

### 2.7. Sample Collection

Placentas were collected at lambing or abortion and examined for evidence of EAE. Placentas were carefully cleared of any attached bedding, oriented to expose the cotyledons and an assessment made of the percentage of area affected by gross pathology, as described previously [47]. Where gross pathology was evident, placental cotyledons were excised using disposable instruments and placed in a sterile bijou for the subsequent preparation of smears and the detection of chlamydial organisms by modified Ziehl–Neelsen (mZN) staining and PCR [43]. Where no gross placental pathology was evident, cotyledons were excised from three different areas of the placenta and combined for mZN and PCR analysis. Cotyledons plus surrounding intercotyledonary membrane were also collected and placed in 10% buffered formalin (BF) for routine histological examination and immunohistochemistry (IHC) to confirm EAE. Three vaginal swabs were taken from each animal at parturition following expulsion of the placenta, for analysis by quantitative real-time polymerase chain reaction (qPCR) to estimate chlamydial load as a measure of bacterial shedding [47]. For any foetuses recovered from suspected non-chlamydial causes of foetal death, samples of brain, lung, heart and liver were placed in 10% BF for histopathological investigation and IHC. Blood samples were collected prior to vaccination and at regular intervals throughout the study for serological analysis by ELISA and for cellular analysis (Figure 1). Ten millilitres of venous blood were collected into vacutainers for the preparation of serum (Becton-Dickinson, Cambridge, UK), while an additional 20 mL were collected into vacutainers containing lithium heparin (Becton-Dickinson) for the cellular studies.

### 2.8. Quantitative Real-Time PCR (qPCR)

The two vaginal swabs collected from each animal following delivery of placentas were vortexed vigorously in 1 mL PBS and centrifuged at 12,500× *g* for 10 min in a standard bench-top microcentrifuge. DNA was extracted from the pellets using a DNeasy^®^ Blood and Tissue Kit (Qiagen Ltd., Crawley, UK) into 200 μL of supplied elution buffer AE, following manufacturer’s instructions. Total DNA was also extracted from approximately 25 mg of placental tissue using a DNeasy^®^ Blood and Tissue Kit and eluted into 200 μL buffer AE as per manufacturer’s instructions. qPCR was carried out on eluted DNA samples, using an ABI Prism 7000 sequence detection system (Applied Biosystems), as described previously [47]. Briefly, the PCR reaction consisted of 12.5 μL of Applied Biosystems™ 2X TaqMan™ Universal PCR Master Mix (Fisher Scientific, Loughborough, UK), 300 nM final concentration of each primer (OmpA forward primer 5′-GCGGCATTCAACCTCGTT-3′ and reverse primer, 5′-CCTTGAGTGATGCCTACATTGG-3′), 250 nM final concentration of fluorescent probe (TaqMan probe, 5′-TGTTAAAGGATCCTCCATAGCAGCTGATCAG-3′) and 1μL swab DNA made up to 25 μL final volume with sterile deionised water. Purified *C. abortus* S26/3 genomic DNA (gDNA) was used as a quantitative standard. The thermal cycling conditions were 50 °C for 2 min and 95 °C for 10 min, followed by 45 cycles of 95 °C for 15 s and 60 °C for 1 min. Each sample was tested in triplicate and results were expressed as the number of *C. abortus* genome copies per µL total swab-extracted DNA.

### 2.9. PCR-Restriction Fragment Length Polymorphism (RFLP) Analysis

All qPCR-positive swab or placental samples from Group 1 were further analysed by PCR-RFLP analysis to confirm that any shedding of chlamydial organisms at parturition were of a vaccine type (vt) in these animals. PCR-RFLP analysis differentiates vt and wild-type (wt) strains of *C. abortus* based on mutations in restriction enzyme sites *Sfc*I, *Hae*III and *Sau3*AI in genes CAB648, CAB153 and CAB636, respectively, in *C. abortus* strain S26/3 [23,48] that ablate cleavage of PCR fragments derived from vt strains. The PCR primers and assay conditions were as previously published [23]. DNA derived from the *C. abortus* 1B vaccine strain and UK wt reference strain S26/3 were used for comparison.

### 2.10. Histopathological Examination and Immunohistochemical (IHC) Analysis

Fixed placental and foetal samples were processed and embedded in paraffin wax as described previously for histopathological and IHC analysis [21]. For histopathological examination, 5 μm serial sections were cut, stained with haematoxylin and eosin. For IHC, sections were labelled with a mouse monoclonal antibody (mAb) to the lipopolysaccharide of *C. abortus* strain S26/3 (mAb 13/4, Santa Cruz Biotechnology, Heidelberg, Germany). Bound antibody was detected using a goat anti-mouse IgG conjugate (Envision™+ System HRP labelled polymer, Dako, Ely, UK), counterstained with haematoxylin and mounted, as previously described [21].

### 2.11. Serological Analysis

Collected venous blood samples were left at room temperature to coagulate and sera collected following centrifugation at 2000× *g* for 15 min. Serum samples were analysed by rOMP90B-3 ELISA, as previously described [46]. Optical densities were normalised using positive and negative control sera and then expressed as a percentage of the positive control using the following formula: [(OD sample − OD negative control)/(OD positive control − OD negative control)] × 100, as previously described [46].

### 2.12. Cellular Analysis

PBMC were isolated from venous blood, counted, adjusted to 2 × 10^6^ cells/mL in Iscove’s Modified Dulbecco’s Medium (IMDM) supplemented with 10% heat-inactivated Foetal Bovine Serum (FBS), 2 mM L-glutamine, gentamicin (50 μg/mL), 100 IU/mL penicillin, 50 μg/mL streptomycin and 50 μM β-mercaptoethanol (Sigma-Aldrich, Dorset, UK) and cultured, according to previously described protocols [49]. To the cells, 100 μL of purified *C. abortus* EB antigen (5 µg/mL) as prepared in Section 2.2, 100 μL of ConA (5 μg/mL; extract from *Concanavalia ensiformis*, ICN Biochemicals, Cleveland, OH, USA) or 100 μL of medium alone were added in quadruplicate wells for each treatment. Antigen-specific recall responses were assessed by analysis of the culture supernatants collected after 96 h for cytokines Interferon-gamma (IFN-γ), Interleukin (IL)-4 and IL-10, as described previously [49]. Supernatants were stored at −70 °C until further analysis. IFN-γ was measured using the indirect ELISA using antibody pairs (mAb clones CC330 and CC302). Quantification was performed by generating a standard curve using known concentrations of recombinant bovine IFN-γ (Endogen-Pierce, Waltham, MA, USA). IL-4 and IL-10 were measured in an indirect ELISA using antibody pairs (mAb clones CC313 and CC314 for IL-4; CC318 and CC320 for IL-10, BioRad Antibody Laboratories, Oxford UK) and quantified using recombinant bovine (rbov) IL-4 of known concentration and recombinant ovine IL-10 of known biological activity [49].

### 2.13. Statistical Analysis

Data on the incidence of abortion and detection of gross infection were modelled using generalised linear models (GLMs) assuming a binomial distribution for the data and a logit link function. Model parameters were estimated by the maximum likelihood method including a bias-reduction correction to accommodate the lack of variability in the outcome within some of the treatment groups. The models included ‘group’ as an explanatory variable and the overall statistical significance of the group effect was assessed using a chi-square statistic.

PCR quantification data were summarised using geometric mean and geometric standard error of the mean (SEM) in better accordance with the characteristics of their distribution (highly right-skewed positive values; geometric statistics obtained from ordinary statistics computed on (log + 1)-transformed data and exponentiated to be expressed in original units). The relative response in the vaccinated groups was statistically compared with the challenge control group by non-parametric rank-based multiple testing using a Dunnett’s [50] type contrast.

Serological (ELISA) and cytokine (IFN-γ and IL-10) responses at different time points were compared between groups using linear mixed models (LMMs) with identity link function and Gaussian errors fitted by restricted maximum likelihood to rank-based inverse normal transformed data. Group, time (either day of gestation or cellular bleed for serological and cytokine data, respectively) and a group:time interaction term were included in the models as fixed-effect factors, whereas animal ID was specified as a random effect. An analogous LMM was fitted to compare antibody response between animals that lambed and aborted in the unvaccinated challenge control group, with pregnancy outcome, time and the interaction between them included as fixed effects and animal ID used as random effect. Significance of the fixed effects was assessed by conditional F-tests. Post hoc pair-wise comparisons between groups used t-tests based on marginal means estimated from the LMM fits.

All statistical analyses were carried out using the R system for statistical computing v4 [51]. Statistical significance was generally assessed at the usual 5% significance level. Where multiple comparisons of groups were conducted, *p*-values were adjusted to control for false discovery rate (FDR) using the Benjamini–Hochberg’s method [52].

## 3. Results

### 3.1. Clinical Outcome of Pregnancy

For the purposes of the results and statistical calculations, ewes were deemed to have aborted if they produced one or more dead foetuses or gave birth to live non-viable lambs that were euthanised on the grounds of animal welfare or which died within 48 h (i.e., neonatal deaths and stillbirths). Abortions were judged to be due to *C. abortus* if chlamydial organisms and/or DNA could be detected by mZN, qPCR or following pathological investigations.

The outcomes of pregnancy for each of the three vaccinated (groups 1–3) and two control groups (groups 4 and 5) are shown in Table 1. For the two experimental vaccine groups (2 and 3), all ewes gave birth to viable live lambs around the expected date of parturition (gestation ranging from 140 to 147 days). For the ewes vaccinated with the commercial live vaccine (group 1), one ewe aborted a single foetus at day 129 of gestation, which was subsequently confirmed to be due to *C. abortus*. Another ewe produced a single lamb which was found dead at day 142 of gestation and still encased in the placental membranes. This lamb death was subsequently shown not to result from *C. abortus*, instead likely resulted from dystocia and asphyxiation as there was clear evidence that the lamb never took a breath.

There were no abortions in any of the negative control animals (group 5) with all animals lambing at the expected time (gestational range of 140–148 days). In the challenge control group 4, 13 of the 25 ewes aborted a total of 20 foetuses (1 × quadruplets, 1 × triplets, 5 × twins and 3 × singles) and 5 non-viable (2 × twins and 1 × single) lambs (gestational range of 122–140 days).

Although there was a single abortion event in the group 1 ewes despite administration of the live vaccine, there was a statistically significant lower abortion rate in this group (and in groups 2 and 3) when compared to the positive control group 4 animals (*p* < 0.001).

### 3.2. Detection of C. abortus Infection

Initial investigation of all the placentas collected from aborted and lambed ewes revealed that gross pathology was evident not only for the ewes that aborted in groups 1 and 4 but also for some of the other ewes that gave birth to apparently healthy lambs (Table 2). In general, for those ewes that aborted, gross pathology was present over the entire placental surface, while for those that lambed, pathology was generally less extensive, although there were a small number of exceptions (Table S1). It was clear from this analysis that the placentas from all but one ewe in group 4 (n = 24/25; 96%) irrespective of having aborted or lambed had evidence of gross infection, while those in groups 1 (n = 4/25; 16%) and 3 (n = 3/24; 12.5%) had a much lower rate of grossly infected placentas. None of the placentas in group 2 had any observable signs of gross pathology. The analysis of placental smears to identify the presence of organisms following mZN staining matched well with the gross pathology results, with organisms detected in a few extra placentas in group 2 where no lesions were evident (Table 2; Table S1), essentially showing a small increase in the sensitivity of detection of infection. Similarly, qPCR of placental samples added yet another level of sensitivity, such that all placentas in challenge control group 4 were positive (n = 25/25; 100%) when taking into account presence of gross pathology, organism and organism DNA (herein referred to as “infection rate”). When looking at the 3 vaccinated groups there was a statistically significant reduction in this infection rate when compared to group 4 (*p* < 0.001): group 1, n = 10/25 (40%) were qPCR positive; group 2, n = 6/26 (23.1%) were qPCR positive; group 3, n = 14/24 (58.3%) were qPCR positive. Overall, group 2 appeared to have the greatest reduction in placental infection. There was no evidence of infection in any of the placentas from the negative control group 5.

Large numbers of *C. abortus* genome copies (0.9–2.0 × 10^6^) were detected on vaginal swabs taken following parturition and the delivery of placentas from most of the aborted and lambed ewes in challenge control group 4, as indicated by the high means presented in Table 2 (also see complete data for individual animals in Table S1). The mean number of organisms detected in swabs (herein referred to as “bacterial load”) calculated as for the vaccinated animals is considerably lower, reflecting the skew of the data towards lower values for many of the animals (as shown in Table S1). Indeed, while 23/25 animals had bacterial loads ranging from 0.5 × 10^3^ to 6.6 × 10^6^ genome copies in group 4 (with the remaining two animals below 48), only three animals had equivalent loads in groups 2 (range of 2 × 10^3^ to 9.2 × 10^4^; remaining 23 animals below 197) and 3 (range of 2.3 × 10^5^ to 2.7 × 10^6^; remaining 21 animals below 87), while 6/25 had equivalent loads in group 1 (range of 0.8 × 10^3^ to 4.3 × 10^6^; remaining 19 animals below 242). Thus, there was a significant reduction in bacterial load excreted following parturition in all three vaccinated groups compared to the challenge control group (*p* < 0.001). Furthermore, although the bacterial load in animals from experimental vaccinate groups 2 and 3 was lower than in animals in the commercial vaccinated group 1, this was not regarded as statistically significant (*p* > 0.287).

### 3.3. Histology and Immunohistochemical Analysis

Histological and IHC analysis was performed on a random selection of placentas and foetuses and where an unusual event occurred, such as the abortion and the lamb found dead wrapped in its membranes, both of which were in group 1. Analysed samples exhibited different degrees of pathology in keeping with clinical outcome. Suppurative necrotising placentitis with vasculitis, observed in the affected placentas, and positive labelling by IHC was diagnostic of *C. abortus* infection and was indistinguishable from the pathology previously reported for this disease [21,47,53]. For the group 1 lamb found dead, the foetal liver showed some evidence of congestion, however, histology and IHC revealed no lesions or labelling typical of EAE in all samples examined.

### 3.4. PCR-RFLP Analysis

Placental tissue and vaginal swab samples from ewes in group 1 that were qPCR positive and exhibited placental gross pathology or were mZN positive were further analysed by PCR-RFLP to determine whether the detected organism was of wild-type or vaccine origin. All samples analysed from ewes that lambed normally were wild-type in origin, resulting from the challenge. However, the animal (Ewe 316D; Table S1) that aborted had evidence of both vaccine type and wild-type strains in both the placenta and on the swabs (Figure S1).

### 3.5. Antibody Responses

**Figure 2:**
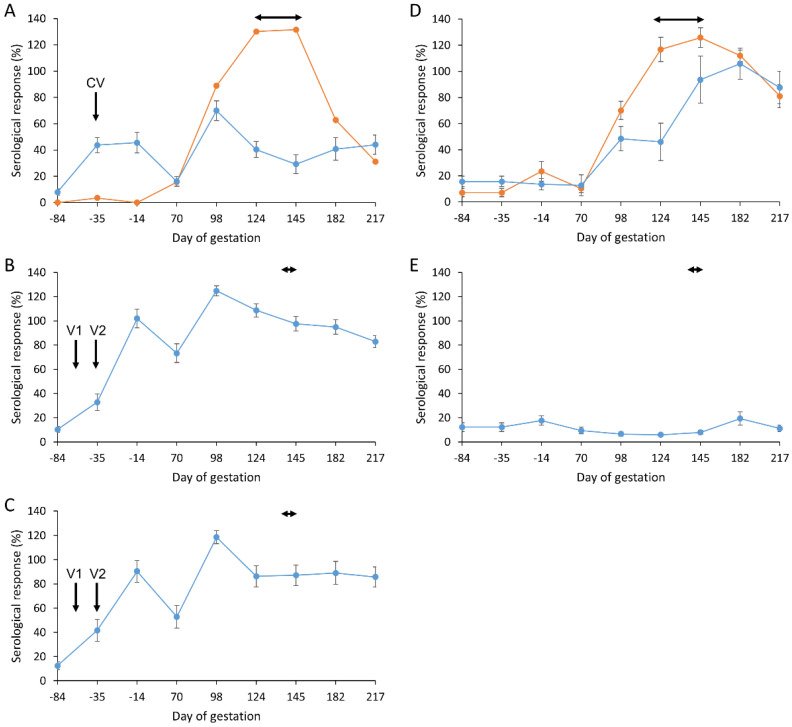
Serological responses following vaccination and challenge with *Chlamydia abortus*. Detection of *C. abortus* antibody in ewes vaccinated (V1 and CV/V2—see Figure 1) with commercial vaccine 1 (**A**) or experimental vaccines 2 (**B**) and 3 (**C**) and challenged at day 70 of gestation with *C. abortus* strain S26/3. Unvaccinated challenged (**D**) and unvaccinated non-challenged (**E**) ewes served as positive and negative control groups. Data are separated into lambed (blue lines) versus aborted (orange lines). Data points represent the arithmetic mean values for each cellular bleed and error bars represent the standard error of that mean (SEM). The 100% is equivalent to an OD450 nm of 2.25. The lambing/abortion period is indicated by the horizontal double-headed arrows.

The mean antibody responses detected for aborted and lambed animals in each of the vaccinated/challenged and control groups are depicted in Figure 2. All ewes were confirmed as seronegative prior to vaccination and mating. A clear antibody response was elicited following vaccination with both the commercial (Figure 2A) and experimental (Figure 2B,C) vaccines in all animals that went on to lamb at parturition. However, the magnitude of response was much greater in animals receiving two doses of the experimental vaccines (V1 and V2 in Figure 2B,C) than in animals receiving a single dose of the commercial vaccine (CV in Figure 2A) (overall mean serological responses of 80.77% and 73.76% in groups 2 and 3, respectively, versus 38.05% in group 1; *p* < 0.001). These antibody responses waned in all three vaccinated groups just prior to challenge but following challenge at day 70 of gestation the responses rapidly increased in magnitude until around day 100–120 and then started to decline or stay relatively constant throughout parturition. This contrasted somewhat with the pattern observed in the unvaccinated challenge control animals (group 4) that lambed where the antibody response increased markedly after challenge and continued to rise throughout parturition (Figure 2D).

Only one animal aborted amongst the vaccinated animals and intriguingly for this single animal, which was in the commercial vaccinated group (group 1) (Figure 2A), there was no detectable antibody response to the vaccine. However, following challenge, the antibody response observed in this animal was much greater than that observed in the animals that lambed and more comparable in magnitude to the unvaccinated challenge control animals.

There was no significant difference observed overall in mean serological response in the unvaccinated challenge control group (Figure 2D) between those animals that lambed and aborted (*p* = 0.265). This probably reflects the fact that all of the animals in this group except one showed strong evidence of heavy infection in the placentas in terms of gross pathology, mZN staining of placental smears or qPCR detection of organisms in placental extracts, irrespective of pregnancy outcome.

All the animals in the negative control group (group 5) (Figure 2E) remained serologically negative throughout the experiment.

### 3.6. Cellular Responses

**Figure 3:**
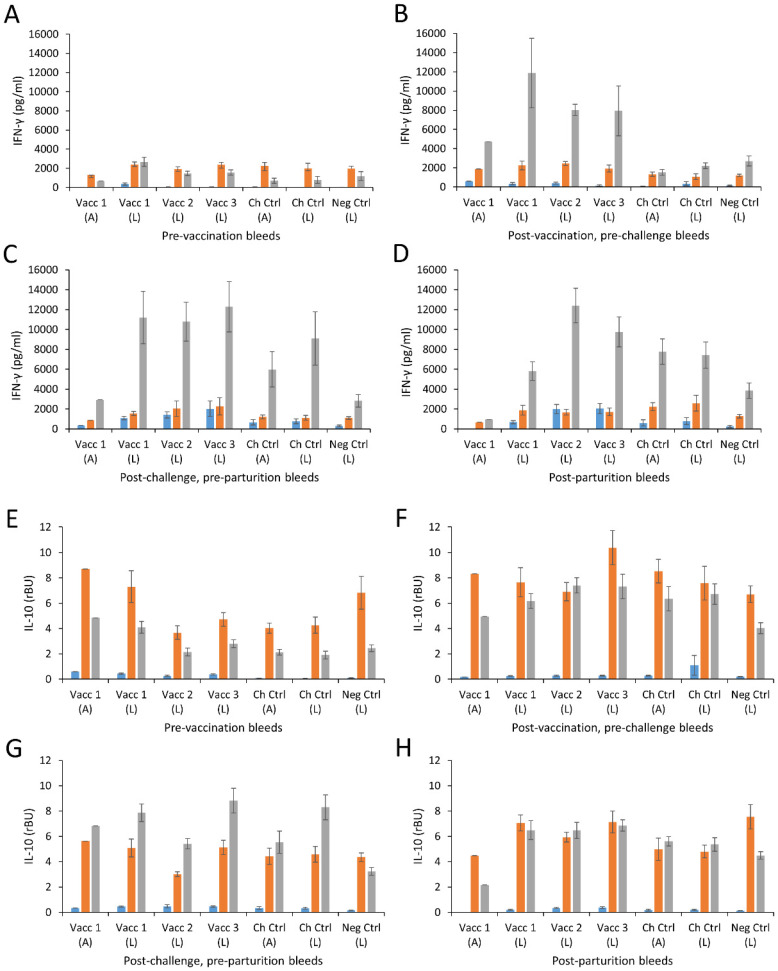
Interferon-γ and IL-10 responses following vaccination and challenge with *Chlamydia abortus*. Peripheral blood mononuclear cells (PBMC) from the animals in the commercial (Vaccine 1) and experimental (Vaccines 2 and 3) vaccine groups were purified from whole blood (as described in Section 2.12 “Cellular Analysis”) collected pre-vaccination (**A**,**E**), post-vaccination/pre-challenge (**B**,**F**), post-challenge/pre-parturition (**C**,**G**) and post-parturition (**D**,**H**) (also see Figure 1). PBMC were set up in lymphocyte stimulation assays in vitro using medium only as an unstimulated cell control (blue bars), the mitogen Concanavalin A (ConA) as a positive control (orange bars) and UV-inactivated *C. abortus* EB antigen (grey bars) for measuring chlamydial antigen-specific stimulation. Antigen-specific recall responses were assessed by analysis of the culture supernatants for cytokines IFN-γ (**A**–**D**) and IL-10 (**E**–**H**). Data for lambed (L) and aborted (A) ewes are presented separately. Data points represent the mean values for each cellular bleed and error bars represent the standard error of that mean (SEM).

Cellular assays were conducted on supernatants generated by stimulation of PBMC with chlamydial EB antigen, as well as with ConA mitogen and unstimulated cells serving as positive and negative controls, respectively. The results are shown in Figure 3 and Figure S2 and are separated according to pregnancy outcome. ConA stimulation induced detectable levels of IFN-γ, IL-10 and IL-4 in all vaccinated and control groups, irrespective of pregnancy outcome. IL-4 data were low/undetectable for antigen restimulation (data shown in Figure S2). The pre-vaccination IFN-γ response to EB antigen was consistent across all groups (Figure 3A). Following vaccination, there was a strong upregulation of chlamydial EB antigen-driven IFN-γ recall responses in animals that lambed from all three vaccine groups (Figure 3B), which were not detected to be statistically different in mean from each other (*p* > 0.116). There was a single animal that aborted in group 1 and although it is not possible to draw statistical comparisons, the magnitude of the IFN-γ response (4720.84 pg/ml) was lower in this sheep than for the other sheep in the same vaccine group (mean response = 11,875.50 pg/mL) or indeed the other vaccinated groups (group 2, mean response = 8018.26 pg/ml; group 3, mean response = 7942.20 pg/mL). This difference was maintained for the duration of the study.

Following challenge infection, the magnitude of the chlamydial EB-induced IFN-γ response was at least maintained if not slightly elevated in the animals that lambed in the vaccinated groups (Figure 3C) and again these results were not statistically different from each other (*p* = 0.844). There were elevated responses in the challenge controls with slightly stronger responses in sheep that go on to lamb than those which aborted, but the difference was not statistically significant in mean (*p* = 0.124). There was no noticeable change to responses in the negative control group at this time point. In the post-parturition sample-point the IFN-γ responses dropped slightly across the vaccine 1 and vaccine 3 groups and challenge controls. This reduction between successive sample points was statistically significant in vaccine 1 group alone (*p* = 0.03; Figure 3D).

The antigen-driven IL-10 responses in the pre-vaccination bleed were broadly similar (Figure 3E). Post-vaccination, elevated responses were observed across all groups irrespective of vaccination status (Figure 3F), although the vaccinated groups and challenge control groups were elevated to a greater extent than the negative control group 5 animals (*p* < 0.0014). The observations of IL-10 in the challenge controls here are pre-challenge and may be a reflection of inter-sample point variability. There was a slight further elevation in some groups following *Chlamydia* challenge irrespective of vaccine status (Figure 3G). Post-parturition there was a maintenance or slight drop in the anti-chlamydial EB IL-10 response (Figure 3H) across the groups except the negative controls. This indicates that both vaccination and challenge contributed to the IL-10 responses observed.

## 4. Discussion

While infections caused by *C. abortus* can be controlled through the use of vaccines, the disease still remains one of the major causes of foetal loss in small ruminants and results in huge economic losses worldwide [4,54,55]. Over the last 60+ years a number of commercialised inactivated vaccines have come and gone for a variety of reasons, including issues with vaccine efficacy and disease outbreaks in vaccinated flocks [15,16,26,56]. Furthermore, the attenuation of the live vaccines has been called into question and these vaccines have been shown to cause disease in some animals [21,22,24,25]. Therefore, there remains a clear need to produce a new efficacious and safer *C. abortus* vaccine, as well as one with increased stability, that results in reduced shedding of infectious organisms at parturition and is economically viable.

In this study we evaluated two subcellular vaccine preparations (COMC and OG-COMC) based on detergent extracted fractions of the *C. abortus* EB and compared them to the commercial vaccine. While studies in mouse models may have a place in the initial evaluation of potential vaccine candidate antigens, mice and sheep clearly differ in terms of immunological response, placentation type [57,58,59,60] and gestational length, as well as other factors, and thus the best model system will always be the natural host animal [61]. Therefore, evaluation of the two experimental vaccines in this study was performed in our established pregnant sheep model [43,47]. While the efficacy of a vaccine can be affected by a number of factors, including the dose of antigen administered, whether the vaccine is administered in a prime-boost approach or in a single inoculation and what adjuvant is used to boost the immune response, it is difficult to address all of these in a single experimental trial. Thus, as a first stage in the evaluation of these two experimental subcellular vaccines we chose to focus our study on the dose of antigen administered using a prime-boost approach. A previous vaccine study at Moredun [34] used 20 μg protein based on a similar preparation of antigen and we have previously determined the MOMP protein content of a single dose of the commercial vaccine of approximately 10^5^ IFU *C. abortus* to contain around 20 μg of MOMP (unpublished). Therefore, this gave us a good starting point in choosing the amount of antigen to use in our experimental vaccines and to ensure that the vaccine would be as economically viable as the current commercial vaccines in terms of growth and preparation. The dose chosen at 20 μg equivalent MOMP protein was then administered in two 10 μg doses with 3 weeks between each dose. Indeed, the effect of this prime-boost approach was clearly evident from the elevated antibody responses observed following the second vaccinations.

At the time the study was conducted we chose to incorporate the Seppic adjuvant Montanide^TM^ ISA 70 VG into our experimental vaccine formulations. This adjuvant at that time was recommended to us by Seppic for eliciting both humoral and cellular responses in our target ruminant species. We note that subsequent to this study that Seppic informed us that they no longer recommended the use of ISA 70 VG in ruminants. This primarily resulted from a study investigating the immunogenicity and tissue reactivity of a *Mycobacterium avium* subsp. *paratuberculosis* inactivated whole cell vaccine, in which the authors concluded that this adjuvant does not induce strong IFN-γ recall responses in sheep in comparison to the newer ISA 61 VG adjuvant [62]. However, in our opinion the data in that study was confounded by the variability in IFN-γ response across the time points they investigated. Furthermore, we believe the data in this study shows that chlamydial antigen-specific IFN-γ recall responses were elicited when using ISA 70 VG in our vaccine formulations.

In this trial, using a simple readout of clinical outcome for evaluation of vaccine efficacy, we found that the two experimental vaccines resulted in no abortion events, while a single abortion occurred in an animal vaccinated with the commercial 1B vaccine. However, there was clear statistically significant difference in all three vaccine groups compared to the challenge control group, showing that all vaccines were effective in reducing the number of abortions. The single abortion event that occurred in the commercial vaccine group was investigated to determine whether disease was a result of vaccine breakdown caused by the challenge strain or whether it resulted from the vaccine strain, as has been previously reported [21,22,23,25]. PCR-RFLP analysis showed that both wild-type and vaccine strains were present in the placenta and on vaginal swabs and that they appeared to be present in approximately equal numbers. This suggests that both strains were probably responsible for the placental infection, gross pathology and for causing the abortion, although it should be noted that all *C. abortus* positive placentas from other animals in this group that lambed normally only had the wild-type strain present.

Clinical outcome is not the only factor that needs to be considered when evaluating the efficacy of a vaccine formulation. The biggest sources of infection responsible for transmission of infectious organisms to naïve animals come from the products of abortion, specifically infected placentas and dead lambs, as well as from the vaginal fluids that follow delivery of lambs and placentas. Therefore, we additionally evaluated the extent of pathology observed in the placenta, the presence of organisms in placental smears and the load of organism in the placentas, as measures of infection and indicators of possible contributory factors in transmission. The results clearly showed that qPCR was the more sensitive method for identifying organisms in the placentas of animals that lambed normally and revealed that the group receiving the COMC vaccine had the lowest detectable presence of organism, compared to the commercial vaccine and OG-COMC vaccine groups (6 versus 10 and 14, respectively), while all placentas in the vaccine control group were found to be positive. However, this approach may be considered to be a ‘blunt instrument’ in terms of trying to correlate positivity with potential for transmission, since a positive qPCR result for a small area of visible gross placental pathology in a placenta where there is no extensive pathology evident or where only low numbers of organisms are present may not be so important in terms of transmission to naïve animals. Nonetheless it is still an indication that vaccine 2 performed better than the other experimental vaccine or indeed the current commercial live vaccine.

Another characteristic of chlamydial abortion is the presence of infectious organisms in the vaginal excretions that follow parturition and the delivery of the placentas, which have also been suggested to play an important role in transmissibility to naïve animals, affecting their reproductive outcomes either in the same lambing season or the subsequent one [45,63,64,65]. The load of pathogen in these excretions clearly plays an important role in this, as does the time taken for these excretions to dry up and lose their infectivity [45]. Therefore, another measure of the efficacy of a successful vaccine comes from it reducing this excretion or “shedding” of organisms post birth. The only caveat of course to this is that qPCR only detects DNA and therefore does not truly detect ‘live’ organism, nonetheless we believe this gives a good indication of infectivity. Previously, we have assessed the level of bacterial shedding by qPCR of vaginal swabs generally taken close to the delivery of the placentas [43]. Results in this study showed that animals in the two experimental vaccine groups (COMC and OG-COMC) had less evidence of bacterial shedding compared to the commercial vaccine group (n = 2 and 3 versus 6, respectively), again suggesting that the COMC vaccine is performing the best.

Antibody responses were more elevated in the ewes that received the two experimental vaccines and lambed, compared to those receiving the commercial vaccine, both following vaccination and following challenge. This may give the impression that antibody is important in terms of protection, however, antibody responses in the animals that aborted in the challenge control group and in the single animal that aborted in the commercial group were more elevated than the antibody titres in the lambed animals in the same group, supporting the view that antibody has little or no protective role at least once placental infection is established and in line with the view that antibody has a greater role in the protective response to re-infection rather than a primary infection [66,67]. Indeed, previous work has shown the importance of a cellular response, in particular an IFN-γ response, in controlling *C. abortus* infections in vivo, while observations were re-confirmed using recombinant ovine IFN-γ on ovine cells in vitro [68,69]. Many cell types contribute to the antigen-specific IFN-γ production elevated in all vaccine groups prior to parturition. This elevation of IFN-γ is consistent with observations from PBMC of sheep vaccinated with the live commercial vaccine (Enzovax) [69]. The effectiveness of the cellular response can be restricted by the presence of counter-regulatory cytokines (IL-4 and IL-10) within the same cultures [70]. Assessment of these cytokines revealed virtually no IL-4 production but some clear and variable antigen-specific IL-10 production in all vaccine groups. This data is consistent with previous studies assessing cellular immunity to sheep experimentally infected with *C. abortus* where elevated IFN-γ and IL-10 was observed [43,49]. Although the focus of this study was evaluation of vaccine efficacy rather than detailed dissection of any protective immune response generated by the candidate vaccines, the analyses conducted here with these signature cytokines highlight the difficulties and complexities in identifying immunological correlates of protection. These correlates are only likely to be defined in the future using multi-parameter systems immunology approaches incorporating humoral and cellular analytes supported by functional read-outs and bioinformatics and will be investigated in greater detail once the final vaccine formulation has been determined and evaluated for commercialisation.

A recent study evaluating two recombinant antigens, macrophage infectivity potentiator (MIP) and chlamydial protease-like activity factor (CPAF), in pregnant sheep found that the separate administration of these antigens did not confer any protection following challenge, while a combination of both antigens resulted in a 50% reduction in abortion rate when compared to the unvaccinated challenge control group [27], suggesting that other antigens are required for protective efficacy. Another study evaluated a new commercial inactivated vaccine (INMEVA^®^, Hipra UK and Ireland Ltd., Nottingham, UK) which achieved a 75% reduction in abortion rate and a 55% reduction in the shedding of organisms when compared to the challenge control group [14]. However, in this latter study there was no comparison to show how the vaccine compared to the current live commercial vaccines. In this study we achieved a much greater reduction in abortion rates, when compared to the unvaccinated challenge control group, of 100% for both experimental vaccines (as a consequence of no abortions occurring) compared to a reduction of 92.3% for the commercial live vaccine. These rates were reduced to 74%, 87.5% and 86.4% for commercial, COMC and OG-COMC vaccines, respectively, when considering reductions in *C. abortus* shedding following parturition, again compared to what was observed in the unvaccinated challenge control group. Nonetheless, the results for both experimental vaccines were improved compared to the current commercial live vaccine, and significantly improved compared to the inactivated vaccine (INMEVA^®^) study. Perhaps this reflects that both experimental vaccines more closely mimic the membrane structure and conformation of proteins present on the surface of the live organism that are important in terms of eliciting the required protective immune response, compared to an inactivated vaccine. Furthermore, they possess all the required antigens to elicit protection that is more difficult to mimic through a subunit vaccine approach. As the soluble OG-COMC vaccine performed less well than the COMC from which it was derived, and involves additional steps in its manufacture, it would appear that the COMC vaccine preparation is worth investigating further to determine its suitability for commercialisation.

## 5. Conclusions

In this study we have taken a first step in the development of a new efficacious safe vaccine against *C. abortus* that has all the benefits of the live vaccines by not having any inactivation step that could potentially alter the antigenicity of surface proteins, while having no ability to grow and cause infection and disease in the host animal. Thus, the vaccine is closer in composition and functionality to that of a live organism-based vaccine and possibly the elicited protective immune response than it is to an inactivated one. The vaccine warrants further evaluation to investigate the efficacy of a single inoculation and to determine if the amount of antigen in each dose of vaccine can be reduced further without compromising efficacy, both of which will make it simpler to administer and more cost effective to produce. The lower the required dose the lower the production costs and corresponding increase in commercial viability.

## Figures and Tables

**Table 1:** The clinical outcome of pregnancy in vaccinated ewes that were subsequently challenged with *Chlamydia abortus* at day 70 of gestation (groups 1–3), in infected control ewes (group 4), or in uninfected control ewes (group 5).

Group	Ewes				Number of Lambs		
	No. Pregnant	No. Lambed (%)	No. Aborted (%)	Mean Gestational Length	Viable	Non-Viable ^1^	Dead
1	25	24 (96)	1 (4)	142	41	0	2 ^2^
2	26	26 (100)	0 (0)	144	47	0	0
3	24	24 (100)	0 (0)	144	39	0	0
4	25	12 (48)	13 (52)	135	23	5	20
5	25	25 (100)	0 (0)	144	43	0	0

^1^ includes neonatal deaths (born live but died within 48 h) and stillbirths. ^2^ includes death of one lamb due to dystocia/asphyxiation.

**Table 2:** Gross placental pathology, detection of *Chlamydia abortus* organisms in placental smears and detection of genomic DNA in vaginal swabs of vaccinated ewes that were challenged with *C. abortus* at day 70 of gestation (groups 1–3), of infected control ewes (group 4), and of uninfected control ewes (group 5).

Group	Pregnancy Outcome ^1^	No. Ewes	Lesions ^2^	mZN ^3^	Placental qPCR ^4^	Swab qPCR ^5^
1	Lambed	24 ^6^	3+, 21−	3+, 21−	9+, 15−	90.7 (1.65)
	Aborted	1	1+	1+	1+	- ^7^
2	Lambed	26	0+, 26−	3+, 23−	6+, 20−	49.13 (1.71)
3	Lambed	24	3+, 21−	3+, 21−	14+, 10−	73.63 (2.2)
4	Lambed	12	11+, 1−	11+, 1−	12+, 0−	43651.42 (3.39)
	Aborted	13	13+, 0−	13+, 0−	13+, 0−	919232.3 (1.78)
5	Lambed	25	25−	25−	25−	12.14 (1.19)

^1^ See Table 1. ^2^ Number of ewes with gross pathological lesions characteristic of *C. abortus* infection evident in one or more placentas. ^3^ Detection of chlamydial organisms following staining of placental smears: +, positive; −, negative. ^4^ Quantitative real-time polymerase chain reaction (qPCR) detection of *C. abortus* DNA in the cotyledons tested by modified Ziehl-Neelsen (mZN). ^5^ Geometric mean (geometric SEM) of the number of *C. abortus* genomes detected per μL total DNA of swab extract. ^6^ Includes lamb that died of dystocia/asphyxiation. ^7^ The summary statistics cannot be computed for a single value.

## Data Availability

Not applicable.

## Supplementary Materials

The following are available online at https://www.mdpi.com/article/10.3390/vaccines9080898/s1, Figure S1: PCR-RFLP analysis of placental and swab samples from ewes vaccinated with the commercial live vaccine, Figure S2: IL-4 responses following vaccination and challenge with *C. abortus*, Table S1: Raw data on clinical outcome, placental gross pathology, mZN and PCR analysis for each experimental group.
