# Supplementary material for: Efficacy of Two Chlamydia abortus Subcellular Vaccines in a Pregnant Ewe Challenge Model for Ovine Enzootic Abortion
Source: Vaccines (Basel). 2021 Aug 13;9(8):898. doi: 10.3390/vaccines9080898 (PMC8402522; doi:10.3390/vaccines9080898)
Supplement: Supplementary file 1 [file vaccines-09-00898-s001.zip › Livingstone et al - Vaccines - Final Version/E30_11_Approval.pdf]

DETAILS OF EXPERIMENTS ON FARM ANIMALS

Number of Experiments	
E	30 / 11
Date:	13JUN11

HO Project Licence Holder **David Longbottom** *Signature* [signature]

Title of Project Licence **Pathogenesis and control of infectious reproductive loss/failure in ruminants and pigs** *PPL* **60/3869**

Licensee responsible for work **David Longbottom** *PIL* **60/8238**

Other licensees involved **Morag Livingstone and Nick Wheelhouse**

Procedure number to be used (see project licence section 19) **19b1**

Short title of experiment **Protective efficacy of inactivated, subcellular and recombinant chlamydial antigens in pregnant sheep**

Animals (Scientific Procedures) Act, 1986. It is a criminal offence to perform any procedure on animals without appropriate Home Office personal and project cover. **BEFORE** submission, all experiments must be endorsed by the Project Licence holder who should retain a copy of this form.

Have all alternatives to animal use been explored? Yes [x] No [ ]

OTHER REGULATORY MATTERS

Does any of the proposed work involve an organism which has been genetically modified? YES [ ] / NO **x**

If YES give GMAG No. [ ]

State that the project conforms to the regulations concerning:

COSHH/H&S	SAPO	Radioisotopes
Yes	NO	NO

STATISTICS

All proposed experiments must be statistically evaluated by BioSS **BEFORE** submission. State if this has been done? YES **x**/ NO [ ]

BioSS Signature: [signature]

COSTINGS (to be completed in consultation with the Clinical Department)

Item				Total	
Animal purchase	175 x 120 + 10 x 500			£ 26000	
Rental	350	x	200	£ 70000	
Maintenance	No. animals	x Daily charge x	No. days	£	NB OVERTIME must be sanctioned and identified
	175	2.4	200	£ 84000	
Other costs	Overtime for Band A/B staff			£ 2000	
Disposal	175 x 50			£ 8750	
**Total costs**				£190750	

BUDGET NO. **GOV SGDLONGBOTTOM** **MSL Contract No:**

Experiments committee only

Approved		Proceed after amendment		Resubmit		Rejected	

Chairman of Expt. Committee Signature	Signed D.P Knox pp J. Fitzpatrick	Date 21 June 2011
