# Supplementary material for: Efficacy of Two Chlamydia abortus Subcellular Vaccines in a Pregnant Ewe Challenge Model for Ovine Enzootic Abortion
Source: Vaccines (Basel). 2021 Aug 13;9(8):898. doi: 10.3390/vaccines9080898 (PMC8402522; doi:10.3390/vaccines9080898)
Supplement: Supplementary file 1 [file vaccines-09-00898-s001.zip › Livingstone et al - Vaccines - Final Version/Figure S1.pdf]

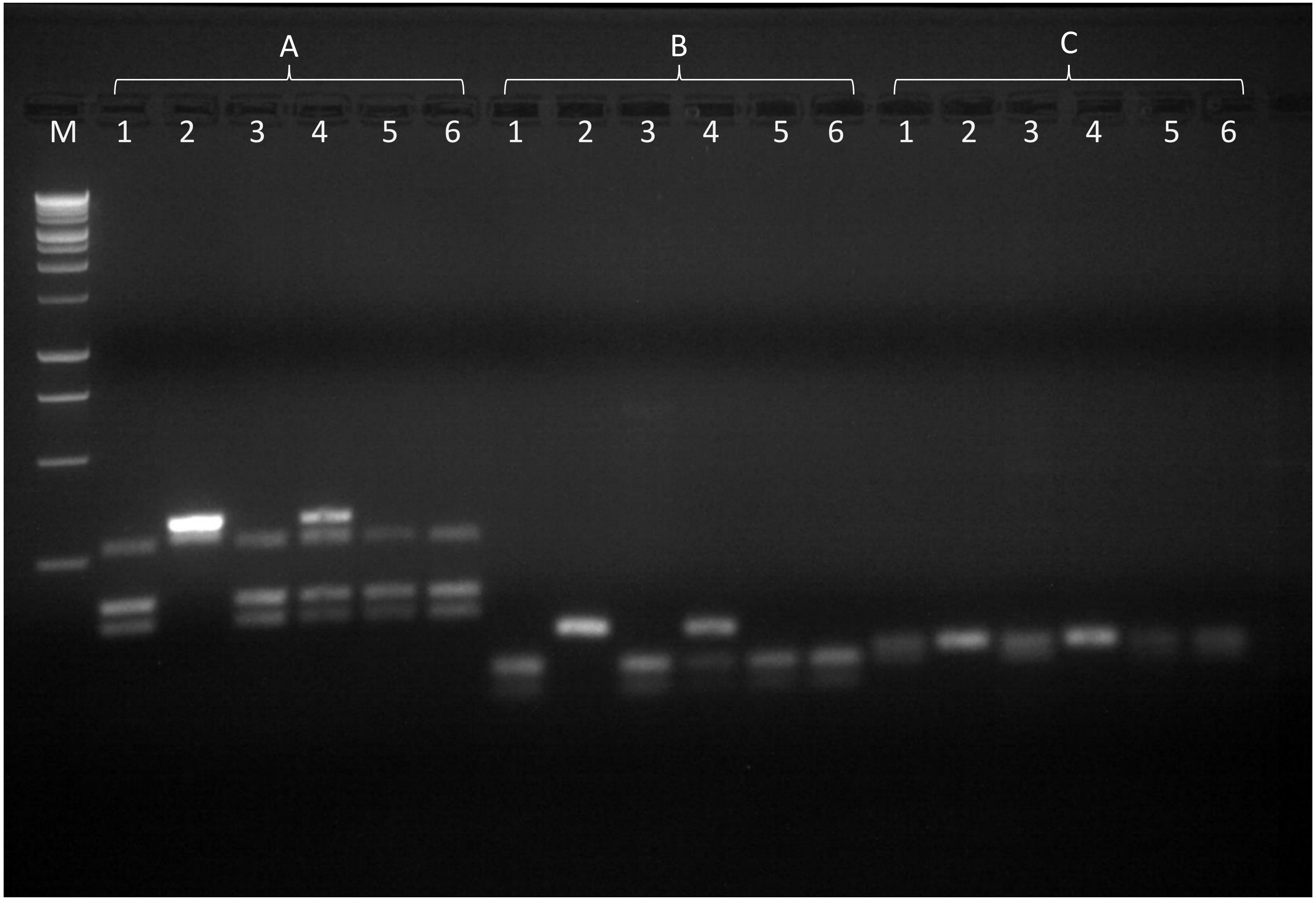

Figure S1. PCR-RFLP analysis of placental and swab samples from ewes vaccinated with the commercial live vaccine. Gel shows restriction endonuclease digestion patterns of CAB153 (digested with SfcI (BfmI)) (A), CAB636 (digested with HaeIII) (B) and CAB648 (digested with Sau3AI (Bsp143I)) (C) PCR amplified fragments of genomic DNA from *C. abortus* strains S26/3 (lane 1), 1B (Cevac vaccine strain) (lane 2), Ewe 203D (lane 3), aborted Ewe 316D (lane 4), Ewe 328D (lane 5) and Ewe 2340E (lane 6) (For individual ewe details see Table S1). Lane M, 1Kb ladder (Promega).
