# Supplementary material for: Efficacy of Two Chlamydia abortus Subcellular Vaccines in a Pregnant Ewe Challenge Model for Ovine Enzootic Abortion
Source: Vaccines (Basel). 2021 Aug 13;9(8):898. doi: 10.3390/vaccines9080898 (PMC8402522; doi:10.3390/vaccines9080898)
Supplement: Supplementary file 1 [file vaccines-09-00898-s001.zip › Livingstone et al - Vaccines - Final Version/Figure S2.pdf]

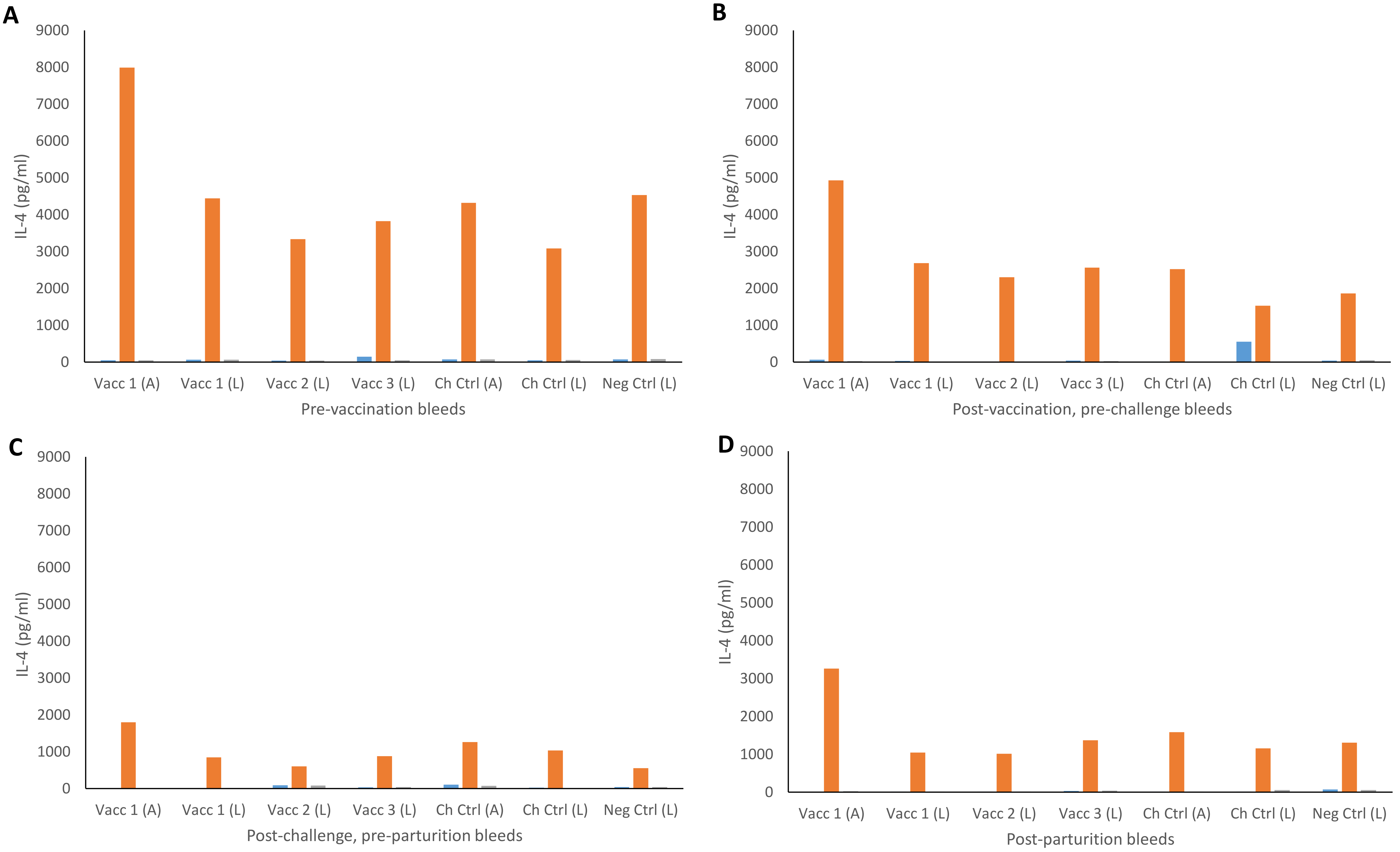

Figure S2. IL-4 responses following vaccination and challenge with C. abortus. Antigen-specific recall responses were assessed by analysis of the culture supernatants for cytokines IL-10. Peripheral blood mononuclear cells (PBMC) from the animals in the commercial (Vacc 1) and experimental (Vacc 2 and Vacc 3) vaccine groups were purified from whole blood (as described in section "2.12. Cellular Analysis") collected pre-vaccination (A), post-vaccination/pre-challenge (B), post-challenge/pre-parturition (C) and post-parturition (D). PBMC were set up in lymphocyte stimulation assays *in vitro* using medium only as an unstimulated cell control (blue bars), the mitogen Concanavalin A (ConA) as a positive control (orange bars) and UV-inactivated *C. abortus* EB antigen (grey bars) for measuring chlamydial antigen-specific stimulation. Data for lambed (L) and aborted (A) ewes are presented separately.
