# Supplementary material for: Efficacy of Two Chlamydia abortus Subcellular Vaccines in a Pregnant Ewe Challenge Model for Ovine Enzootic Abortion
Source: Vaccines (Basel). 2021 Aug 13;9(8):898. doi: 10.3390/vaccines9080898 (PMC8402522; doi:10.3390/vaccines9080898)
Supplement: Supplementary file 1 [file vaccines-09-00898-s001.zip › Livingstone et al - Vaccines - Final Version/Table S1.pdf]

Group 1: Cevac Chlamydia

				Macroscopic examination				mZN				PCR					
Ewe No	Lambed (No)	Aborted (No)	L/A	P1	P2	P3	P4	P1	P2	P3	P4	Typing	Swabs	P1	P2	P3	P4
203D	2	0	L	10	0			+++	+++			wt	12	3900000	494200		
215D	1	0	L	0				-					14	37.71			
221D	2	0	L	0	0			-	0				43	69.12	0		
283D	2	0	L	0	0			-	-				158	124.69	126.88		
316D	0	1	A	100				++++				vt + wt	4340000	2830000			
328D	2	0	L	25	NF			+	NF			wt	3056	42183.82	NF		
1100J	2	0	L	0	0			-	-			wt	73	306.29	617.94		
2295E	1 *	0	L	0				-					812	54.94			
2304E	1	0	L	0				-					46	48.48			
2305E	2	0	L	0	0			-	-				17	105.66	27.94		
2311E	1	0	L	0				-					13	516.6			
2312E	2	0	L	0	0			-	-				34	46.51	43.66		
2313E	3	0	L	0	0	0		-	-	-			53	49.5	32.88	18.94	
2322E	2	0	L	0	0			-	-				21	0	14.81		
2333E	1	0	L	0				-					1544	0			
2340E	1	0	L	85				++++				wt	370000	5820000			
2389E	2	0	L	0	NF			-	NF			wt	14	0	NF		
3641G	2	0	L	0	0			-	-				29	361.31	29.99		
3645G	2	0	L	0	0			-	-				26	0	0		
3701G	2	0	L	0	0			-	-				13	18.41	12.48		
3719G	2	0	L	0	0			-	-				16	0	16.71		
5348G	2	0	L	0	NF			-	NF				959	121.4			
5388G	2	0	L	0	0			-	-				8	11.7	0		
5389G	2	0	L	0	NF			-	NF				242	19.14	NF		
5425G	2	0	L	0	0			-	-				223	32.36	0		
	40	1	24														
			1														

* found dead in membranes; no bacteriological or pathological evidence of EAE.

Group 2: COMC

Ewe No	Lambed (No)	Aborted (No)	L/A	Macroscopic examination				mZN				PCR				
				P1	P2	P3	P4	P1	P2	P3	P4	Swabs	P1	P2	P3	P4
361D	2	0	L	0	0			-	-			34	3.98	0		
372D	2	0	L	0	0			-	-			90	7.44	4.41		
434D	2	0	L	0	0			-	-			19	0	0		
445D	2	0	L	0	0			-	-			106	0	15.19		
454D	3	0	L	0	0	0		-	-	-		24	0	0	0	
462D	2	0	L	0	0			-	-			29	31.13	13.5		
2058E	1	0	L	0				-				37	3.04			
2348E	1	0	L	0				-				29	0			
2367E	3	0	L	0	0	NF		-	-	NF		0	5.19	0	NF	
2370E	2	0	L	0	0			-	-			44	435.03	8.25		
2379E	2	0	L	0	0			-	-			25	8.1	10.7		
2384E	3	0	L	0	0	NF		-	-	NF		0	10173.31	5083.62	NF	
2386E	2	0	L	0	0			-	-			2010	5.22	52.78		
2399E	2	0	L	0	0			-	-			181	296.11	9.11		
2470E	1	0	L	0				-				18	0			
2481E	2	0	L	0	NF			-	NF			71	3.77	0		
2492E	1	0	L	0				+				0	68820.93			
2569E	1	0	L	0				+				57117	7733.82			
3722G	2	0	L	0	0			+++	++			92264	7.9	0		
3741G	2	0	L	0	0			-	-			70	142974.5	116503		
3746G	2	0	L	0	0			-	-			17	7.54	0		
3774G	2	0	L	0	0			-	-			35	0	11.62		
5430G	1	0	L	0				-				35	3.59			
5504G	1	0	L	0				-				197	0			
5509G	2	0	L	0	NF			-	NF			1	15.47			
5511G	1	0	L	0				-				7	49.13			
	47	0	26													
			0													

Group 3: OG-MOMP

Ewe No	Lambed (No)	Aborted (No)	L/A	Macroscopic examination P1	P2	P3	P4	mZN P1	P2	P3	P4	PCR Swabs	P1	P2	P3	P4
466D	2	0	L	0	0			-	-			6	31.56	27.39		
471D	1	0	L	0				-				10	27.52			
473D	1	0	L	0				-				0	31.56			
479D	3	0	L	0	0	0		-	-	-		21	24.48	200.77	23.79	
503D	2	0	L	0	0			-	-			40	100.58	59.11		
525D	2	0	L	0	0			-	-			87	67.45	54.39		
588D	1	0	L	20				++++				1860000	1990000			
608D	2	0	L	40	0			+++	++			2720000	519024.9	65633		
2494E	2	0	L	0	0			-	-			9	297.52	186.26		
2497E	1	0	L	0				-				79	189.85			
2502E	2	0	L	0	0			-	-			5	66.45	165.11		
2548E	2	0	L	0	NF			-	NF			7	901.17	NF		
2553E	2	0	L	0	0			-	-			6	151.68	53.73		
2568E	1	0	L	100				+++				231125	1900000			
2571E	1	0	L	0				-				79	189.49			
2639E	1	0	L	0				-				26	143.96			
3775G	2	0	L	0	0			-	-			59	0	0		
3801G	2	0	L	0	0			-	-			61	20.45	6.02		
3834G	1	0	L	0				-				56	45.3			
3841G	2	0	L	0	0			-	-			25	123.22	31.06		
5532G	1	0	L	0				-				11	8.86			
5533G	1	0	L	0				-				3	17.91			
5543G	2	0	L	0	0			-	-			20	884.49	21.4		
5616G	2	0	L	0	0			-	-			27	21.4	11.2		
	39	0	24													
			0													

Group 4: Challenge Control

Ewe No	Lambed (No)	Aborted (No)	L/A	Macroscopic examination P1	P2	P3	P4	mZN P1	P2	P3	P4	PCR Swabs	P1	P2	P3	P4
76D	1	0	L	1				+++				48	8962.34			
122D	0	4	A	100	100	100	NF	++++	++++	++++	NF	2730000	955686.1	6190000	1040000	NF
131D	0	3	A	100	100	75		+++	+++	+++		560447	450079.8	59446.8	3420000	
1065D	1	0	L	10				++++				2690000	3670000			
1087D	2	0	L	5	NF			+++	50			2383	589006.8	NF		
1116D	0	2	A	100	100			++	+++			968330	75131.09	1810000		
2092E	0	2	A	100	100			+++	++++			1620000	183552.7	1.4E+07		
2093E	0	1	A	100				+++				383942	1530000			
2097E	3	0	L	90	60	50		+++	+++	+++		4280000	1960000	8120000	2180000	
2113E	0	2	A	100	70			+++	++			2790000	2790000	710139		
2246E	0	2	A	100	80			+++	+++			3540000	381150.1	2050000		
2249E	2	0	L	50	NF			++	NF			1870000	no sample	NF		
2266E	0	2 *	A	100	NF			++	NF			6590000	306795.6	NF		
2283E	1	0	L	100				++				134613	154960.2			
2285E	1	1 **	A	90	NF			++	NF			1490000	1680000	NF		
4461G	0	2 ***	A	50	50			+++	+++			2940000	797733	2100000		
4829G	1	0	L	50				+++				118101	1110000			
5043G	1	1	A	100	100			++++	++			998702	2030000	177573		
5153G	1	0	L	80				+++				1200000	381671.1			
5187G	0	2	A	50	100			++	++++			851802	66430.29	1450000		
5276G	2	0	L	0	5			-	+++			42097	1741.28	679085		
5290G	1	0	L	50				+++				1020000	130309.3			
5343G	2	0	L	20	10			+++	+++			504	793136.2	621677		
5724G	2	1	A	0	NF	20		++	NF	++		1496	117209.6	NF	554696	
5778G	2	0	L	0	0			-	-			45	192.87	34		
	23	20	12													
			13													

* born weak; both died within 24 hrs
** born weak; euthanised 37hrs later
*** born weak; euthanised after 11hrs

Group 5: Negative Control

Ewe No	Lambed (No)	Aborted (No)	L/A	Macroscopic examination P1	P2	P3	P4	mZN P1	P2	P3	P4	PCR Swabs	P1	P2	P3	P4
91D	2	0	L	0	0			-	-			9	0	0		
1077D	2	0	L	0	0			-	-			19	0	0		
1086D	2	0	L	0	0			-	-			30	19.54	0		
1088D	2	0	L	0	0			-	-			9	0	0		
1092D	1	0	L	0				-				23	0			
1093D	2	0	L	0	0			-	-			24	0	0		
1120D	1	0	L	0				-				6	77.94			
2055E	2	0	L	0	0			-	-			15	0	0		
2150E	1	0	L	0				-				13	0			
2161E	2	0	L	0	0			-	-			53	0	0		
2195E	2	0	L	0	0			-	-			15	0	0		
2202E	2	0	L	0	0			-	-			13	0	0		
2294E	2	0	L	0	0			-	-			8	0	0		
3375G	1	0	L	0				-				0	0			
3443G	1	0	L	0				-				9	0			
3553G	2	0	L	0	0			-	-			9	25.48	0		
3599G	1	0	L	0				-				10	0			
3607G	2	0	L	0	0			-	-			12	0	0		
3620G	1	0	L	0				-				0	0			
3855G	2	0	L	0	0			-	-			12	0	16.23		
4956G	3	0	L	0	0	NF		-	-	NF		15	0	0	NF	
4982G	2	0	L	0	0			-	-			23	15.09	0		
5057G	2	0	L	0	0			-	-			28	14.58	0		
5344G	2	0	L	0	0			-	-			11	0	0		
5345G	1	0	L	0				-				7	0			
	43	0	25													
			0													
